# Differential phenotypes of memory CD4 and CD8 T cells in the spleen and peripheral tissues following immunostimulatory therapy

**DOI:** 10.1186/s40425-017-0235-4

**Published:** 2017-04-18

**Authors:** Gail D. Sckisel, Annie Mirsoian, Christine M. Minnar, Marka Crittenden, Brendan Curti, Jane Q. Chen, Bruce R. Blazar, Alexander D. Borowsky, Arta M. Monjazeb, William J. Murphy

**Affiliations:** 1 Department of Dermatology, University of California, Davis School of Medicine, Sacramento, CA USA; 2 Earle A. Chiles Research Institute, Robert W. Franz Cancer Center, Providence Portland Medical Center, Portland, OR 97213 USA; 3 The Oregon Clinic, 4805 NE Glisan St, Portland, OR 97220 USA; 4 Department of Pathology and Laboratory Medicine, Center for Comparative Medicine, University of California, County Road 98 & Hutchison Drive, Davis, CA 95616 USA; 5 Department of Pediatrics, Division of Blood and Marrow Transplantation, University of Minnesota Cancer Center, MMC 366 Mayo, 8366A, 420 Delaware Street SE, Minneapolis, MN 55455 USA; 6 Department of Radiation Oncology, University of California, Davis School of Medicine, Comprehensive Cancer Center, 4501 X Street, G-140, Sacramento, CA CA 95817 USA; 7 Department of Internal Medicine, University of California, Davis School of Medicine, Sacramento, CA USA

**Keywords:** Immunotherapy, Cancer, PD-1, Bystander Activation, CD8, NKG2D

## Abstract

**Background:**

Studies assessing immune parameters typically utilize human PBMCs or murine splenocytes to generate data that is interpreted as representative of immune status. Using splenocytes, we have shown memory CD4-T cells that expand following systemic immunostimulatory therapies undergo rapid IFNg-mediated activation induced cell death (AICD) resulting in a net loss of total CD4-T cells which correlates with elevated PD-1 expression. This is in contrast to CD8-T cells which expand with minimal PD-1 upregulation and apoptosis. In this study we expand upon our previous work by evaluating CD4 and CD8-T cell phenotype and distribution in peripheral organs which are more representative of immune responses occurring at metastatic sites following immunotherapy.

**Methods:**

Phenotypic assessment of T cells in both lymphoid (spleen and LN) as well as peripheral organs (liver and lungs) in control and immunotherapy treated mice was performed to survey the impact of location on memory phenotype and activation marker status. Peripheral blood from patients undergoing systemic high dose IL-2 was also assessed for expression of PD-1 and memory phenotype.

**Results:**

Here we reveal that, similar to what occurs in the spleen and lymph nodes, CD4-T cell numbers decreased while CD8-T cells expanded at these peripheral sites. In contrast to having differential expression of PD-1 as occurs in the spleen, both CD4 and CD8-T cells had significantly elevated levels of PD-1 in both the liver and lungs. Further analysis correlated PD-1 expression to CD62L^low^ (T effector/effector memory,T_E/EM_) expression which are more prevalent in CD4-T cells in general as well as CD8-T cells in peripheral organs. Similar elevated PD-1 expression on T_E/EM_ cells was observed in patients undergoing systemic high-dose IL-2 therapy.

**Conclusions:**

These data highlight PD-1 expressing and/or T_E/EM_ subsets of T cells in circulation as more representative of cells at immune sites and underscore the importance of valuation both in lymphoid as well as target organs when making determinations about immune status.

**Trial registration:**

ClinicalTrials.gov NCT01416831. Registered August 12, 2011.

**Electronic supplementary material:**

The online version of this article (doi:10.1186/s40425-017-0235-4) contains supplementary material, which is available to authorized users.

## Background

Recent technological advances in microscopy, cytometry, and advanced molecular techniques have facilitated the evaluation of immune parameters across countless animal models and disease states. These advances have furthered our understanding of how various immune cells function and interact with one another, their roles in different diseases and disorders, as well as their responses to therapies. Often times, systemic parameters such as peripheral blood or lymphoid organs (i.e., spleen, LNs, etc.) are used as surrogates for understanding both what is occurring within the organism as a whole as well as what is occurring at effector sites. This is particularly the case when a systemic agent is administered or when local samples of effector sites aren't easily accessible for complex analysis, such as during clinical trials with human patients in which peripheral blood is the standard for immune monitoring. Whether this practice is actually representative of what is occurring at local sites is debatable. Studies by our group have shown that during systemic cancer immunotherapy, regulatory T cells expand systemically in the spleen and lymph nodes but actually decrease in the tumor itself, thereby minimizing any negative biological effect on therapeutic outcome [1]. Furthermore, in many murine viral studies, the practice of studying effector sites has almost become standard.

Our lab has previously described the presence of a unique, antigen non-specific function of conventional memory CD8 T cells that can occur during periods of heightened immune stimulation such as cancer immunotherapy or viral infection [2–6]. Under these conditions, memory CD8 T cells can be activated purely by exposure to elevated cytokines causing them to expand and upregulate markers such as NKG2D which can confer the ability to respond to target cells that are inappropriately expressing stress ligands instead of through recognition of cognate antigen in the context of MHC, consistent with alternative bystander activation. Importantly, we have shown that these bystander activated memory CD8 T cells do not upregulate markers consistent with TCR engagement, namely CD25 and PD-1. In contrast, CD4 T cells activated under the same conditions undergo proliferation which is heavily coupled with apoptosis resulting in a net insignificant expansion of this cell type. This apoptosis was shown to be IFNg dependent [7] and thought to be occurring through differential expression of PD-1 on CD4 T cells following cytokine-induced, antigen-independent stimulation [8]. While we have exhaustively characterized these opposing roles in both CD4 and CD8 T cells following systemic immunotherapy, the majority of our conclusions have been drawn from data derived from secondary lymphoid organs (i.e., spleen and lymph nodes) and not in the periphery such as the tumor or metastatic sites.

Here we show that during treatment with immunostimulatory therapies for cancer, the bystander expansion and activation phenotype of CD8 T cells varies greatly by organ. Similar to our previous studies we saw that CD8 T cells expanded in both lymphoid and peripheral organs whereas CD4 T cells did not significantly expand in numbers in either lymphoid or peripheral organs because in addition to proliferating, they also were undergoing apoptosis. Interestingly, we noticed that among CD8 T cells, while the expansions were comparable across organs, we noted that the phenotypes of these expanded cells varied in the peripheral organs compared to the lymphoid, as peripheral organs had elevated NKG2D, PD-1, and KLRG1. In contrast, CD4 T cell phenotypes were relatively consistent across all organs. We show that the composition of the memory/activated (CD44^high^) at a given site weighs heavily on the activation marker expression at that site with those being more rich in the effector/effector memory T cell subsets having elevated expression of activation markers across the board. Importantly, PBMC samples from patients receiving systemic high dose IL-2 therapy express elevated levels of PD-1 on the T_E/EM_ subset as well. In summary, these data highlight the critical need to assess immune phenotype and function not only in lymphoid organs, but direct sites of inflammation in order to get an accurate picture of what is occurring locally. Furthermore, it suggests the systemic effector/effector memory population may directly correlate with the phenotype of the cells at peripheral sites.

## Methods

### Mice

Female 8-12 week old female C57BL/6 or BALB/c mice were purchased from the animal production area at the National Cancer Institute (NCI-APA, Frederick, MD). FVB mice were purchased from Charles River Laboratories. The MIN-O tumor model was set up as previously described [9]. All mice were housed in the animal facilities at the University of California, Davis under specific pathogen-free conditions and studies were approved by the UC Davis Institutional Animal Care and Use committee.

### In vivo antibodies and reagents

The agonistic anti-mouse CD40 antibody (FGK115.B3) was generated as previously described [10]. Recombinant human interleukin-2 (rhIL2; Teceleukin, Roche, Germany) was provided by the National Cancer Institute (NCI, Frederick, MD). Rat IgG (Jackson ImmunoResearch Laboratories Inc, West Grove, PA) was used as a control for anti-CD40.

### Immunotherapeutic regimen

Mice were administered agonistic anti-CD40 and rhIL-2 as previously described [7]. Briefly, agonistic anti-CD40 or rat IgG (Jackson Immunoresearch) was administered for 5 days (days 0-4). rhIL-2 or PBS alone was administered twice per week for 2 weeks.

### Human high dose IL-2 trial

Blood samples were obtained from patients with metastatic melanoma enrolled in a randomized Phase II trial and receiving high-dose IL-2 alone as previously described [11]. Patients began treatment on a Monday (day 1) and high-dose IL-2 was administered at 600,000 IU/kg by i.v. bolus infusion given every 8 h for 14 planned doses depending on how well the IL-2 was tolerated. The median number of doses in this cycle of the study was 11. Blood samples were obtained at baseline, day 2, and day 8. The day 8 data will vary from patient to patient depending on how many doses that they tolerated. Peripheral blood mononuclear cells (PBMCs) were cryopreserved from Ficoll-separated blood and stored at -170C. Signed informed consent was obtained before enrollment. The study was approved by the Providence Health System Regional Institutional Review Board, Oregon.

### Antibodies for flow cytometry

All antibodies were purchased commercially and tested for optimal dilution in house based on lot, clone, and vendor. Antibodies purchased from BD Pharmingen included purified anti-mouse CD16/32 (Fc block), PE-Cy5 conjugated anti-human CD45RO, PE conjugated anti-human PD-1, and allophycocyanin (APC)-Cy7 conjugated anti-mouse CD25. Antibodies purchased from eBiosciences included FITC conjugated anti-mouse PD-1, PE-Cy7 conjugated anti-mouse CD62L and NKG2D, and PE-Cy5 conjugated anti-mouse CD62L. Antibodies purchased from Biolegend (San Diego, CA) include pacific blue conjugated anti-mouse CD44, brilliant violet (BV) 421 conjugated anti-human CD45RA, BV605 conjugated anti-mouse CD8, BV711 conjugated anti-mouse CD4 and anti-human CD4, BV785 conjugated anti-mouse CD3, FITC conjugated anti-human CD62L, and PE-Cy7 conjugated anti-human CD8.

### Flow cytometry

In general, 10^6^ cells for surface only stains or 2x10^6^ cells for stains investigating intracellular antigens were stained in round bottom 96 well plates. Surface antibodies were diluted with staining buffer (1% FBS, 1 mM EDTA, and 0.02% NaN_3_ in PBS) into cocktails containing Fc block (purified anti-mouse CD16/32, BD Pharmingen, San Diego, CA) and added to cells at 45 μl per sample. Cells were washed and resuspended in staining buffer for analysis within 24 h. Data were collected using a BD Fortessa instrument running FACS DIVA software. Data were analyzed using FlowJo v10 (TreeStar, Ashland, OR).

### Data analysis and statistics

Statistical analysis was performed using Prism software (GraphPad Software Inc.). Data were expressed as mean ± SEM. For analysis of three or more groups, the non-parametric one or two-way ANOVA test (where appropriate) was performed with the Bonferroni post-test. Analysis of differences between two normally distributed test groups was performed using the Student’s *t*-test. Welch’s correction was applied to Student’s *t*-test data sets with significant differences in variance. * *P* < 0.05, ** *P* < 0.01, *** *P* < 0.001.

## Results

### Systemic agonistic cancer immunotherapy induces differential expansion of CD4 and CD8 T lymphocytes in lymphoid and peripheral organs

Combination of anti-CD40 with IL-2 has been shown to induce delayed growth and regression across several murine tumor models [6, 7, 10]. Similar to published data using cell line tumor models, treatment of the mammary intraepithelial neoplasia-outgrowth (MIN-O) model [9], a tissue transplant line, with anti-CD40 and IL-2 immunotherapy (IT) led to significant anti-tumor responses (*P* = 0.0057) including regression in >50% of the treated mice (Additional file 1: Figure S1A). Previous studies have shown these anti-tumor responses to be due to CD8 T cells therefore we assessed T cell phenotype in the spleen as well as within the tumor and lungs (a common metastatic site for many different tumor models). While we noted therapy generally induced CD8 expansion across all organs, we noted some differences in CD8 T cell memory phenotype across organ sites (Additional file 1: Figure S1B-C).

**Fig. 1:**
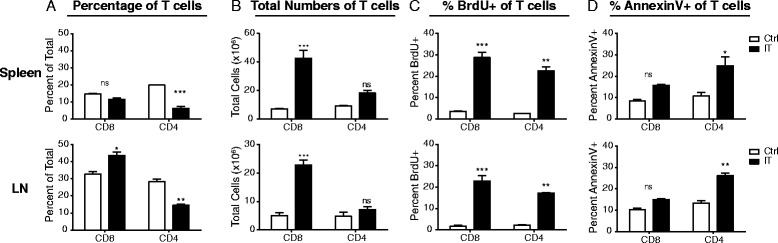
CD4 and CD8 T cells have differential proliferative and apoptotic responses to immunostimulatory therapies in lymphoid organs. Mice were treated with anti-CD40/IL-2 immunotherapy and assessed for various immune parameters on day 12 of treatment in lymphoid (spleen or LN) organs. Percentage (**a**) and total numbers (**b**) of CD4 (Foxp3^-ve^) and CD8 T cells in lymphoid organs. Percentage of proliferating (**c**), as assessed by BrdU, and apoptotic (**d**), as assessed by surface Annexin V expression, of CD4 (Foxp3^-ve^) and CD8 T cells in lymphoid organs. These data are representative of 2-5 independent experiments with 3 mice per group. Data are presented as mean ± SEM. Statistics were derived using ANOVA with Bonferroni’s post-test, *P < 0.05, **P < 0.01, ***P < 0.001, ns: *P* > 0.05

**Fig. 2:**
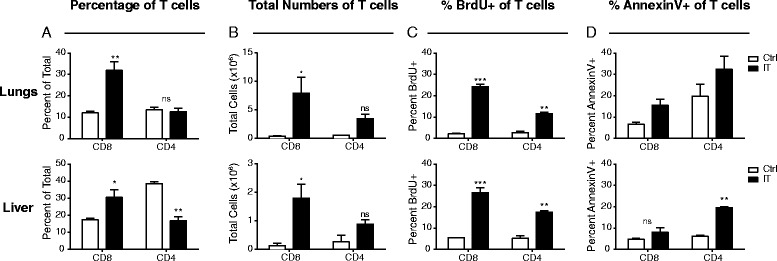
CD4 and CD8 T cells have differential proliferative and apoptotic responses to immunostimulatory therapies in peripheral organs. Mice were treated with anti-CD40/IL-2 immunotherapy and assessed for various immune parameters on day 12 of treatment in peripheral (lungs or liver) organs. Percentage (**a**) and total numbers (**b**) of CD4 (Foxp3^-ve^) and CD8 T cells in peripheral organs. Percentage of proliferating (**c**), as assessed by BrdU, and apoptotic (**d**), as assessed by surface Annexin V expression, of CD4 (Foxp3^-ve^) and CD8 T cells in peripheral organs. These data are representative of 2-3 independent experiments with 3 mice per group. Data are presented as mean ± SEM. Statistics were derived using ANOVA with Bonferroni’s post-test, *P < 0.05, **P < 0.01, ***P < 0.001, ns: *P* > 0.05

We and others have previously shown that strong immunostimulatory therapies for cancer induce potent proliferation of memory (CD44^high^) CD4 and CD8 T cells in the spleen and lymph nodes [6]. It was also observed that CD4, but not CD8, T cells also undergo activation induced cell death in an interferon(IFN)-γ dependent fashion resulting in insignificant overall expansion of CD4 T cells by numbers in these same organs compared to baseline [7]. These data were generated using lymphoid organ readouts. However, in light of phenotypes observed in the MIN-O bearing, immunotherapy treated mice, the expansion, activation, and apoptosis of activated T cells may be differentially affected in the peripheral tissues. Therefore, we sought to further characterize and compare T cell activation in peripheral organs (where the primary tumor and/or metastatic lesions may reside) and secondary lymphoid organs (which are often surveyed during immunotherapeutic studies to assess mechanisms of action). We evaluated CD8 and CD4 T cell (Foxp3^neg^) frequency, expansion, and apoptosis systemically in both lymphoid and peripheral organs. Consistent with previous reports by our group, while not significantly altering their overall frequency (Fig. 1a), anti-CD40/IL-2 immunotherapy resulted in significant expansion in total numbers of CD8 T cells in the spleens and lymph nodes (Fig. 1b). In line with increases in total CD8 numbers, the frequency of CD8 T cells that incorporated bromodeoxyuridine (BrdU) in vivo was significantly expanded and the proportion of apoptotic cells as assessed by extracellular Annexin V expression was not significantly different from controls (Fig C-D). In contrast, total CD4 T cell frequency decreased and numbers did not change significantly compared to controls within the same organs (Fig. 1a-b). While CD4 T cells were expanding as assessed by BrdU incorporation, a significant proportion of them were going through apoptosis as well (Fig. 1c-d) resulting in a net insignificant change in total numbers. These data were in line with what was previously observed [7]. When we assessed non-lymphoid organs including lungs and liver, we saw similar trends in both CD4 and CD8 T cells, namely that CD8 T cells were expanding and surviving across all organs following IT (Fig. 2a-b) whereas CD4 T cells (Foxp3^neg^) were expanding and concurrently going through apoptosis to a similar extent resulting in insignificant changes to both their frequencies and numbers (Fig. 2c-d) in the periphery.

### T cell memory phenotypes vary between secondary lymphoid organs and peripheral non-lymphoid tissues in CD8 T cells but not CD4 T cells following IT

In mice, CD4 and CD8 T cells can be further categorized into memory and naïve phenotypes based on CD62L (L-selectin) and CD44 expression with the CD44^low^CD62L+ population considered naïve (T_N_), CD44^high^CD62L+ population considered central memory (T_CM_), and the CD44^high^CD62L^neg^ population considered effector and/or effector memory (T_E/EM_). It is known that CD4 and CD8 T cells differ in their distribution of these subsets in lymphoid and peripheral organs. While naïve frequencies within CD4 and CD8 populations remain relatively similar, the CD44^high^ population is more central memory skewed in CD8 T cells and effector memory skewed in CD4 T cells in a resting organism [12, 13]. However, in the peripheral organs, tissue resident T cells within both the CD4 and CD8 T cell subsets are predominantly of the effector memory phenotype [14].

**Fig. 3:**
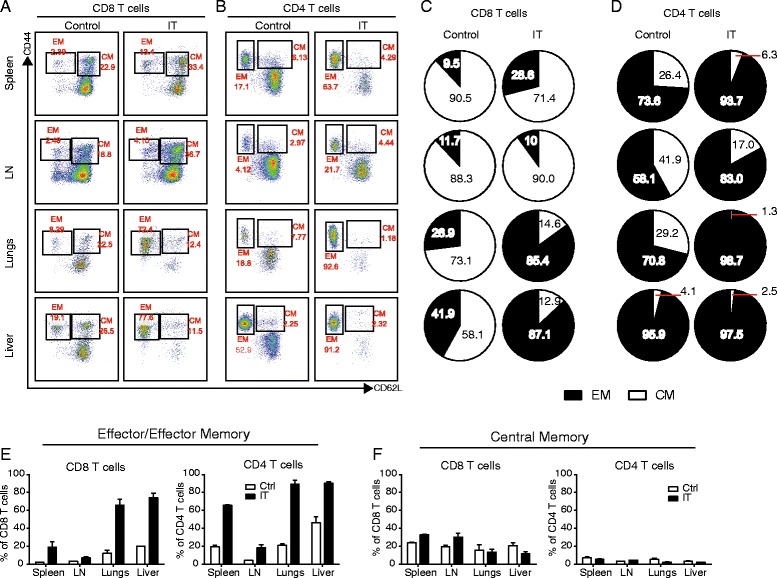
T cell memory phenotype differs in lymphoid and peripheral organs following immunotherapy. Mice were treated with anti-CD40/IL-2 immunotherapy and assessed for various immune parameters on day 12 of treatment in lymphoid (spleen or LN) or peripheral (lungs or liver) organs. **a**-**b** Representative dot plots of CD44 vs CD62L expression in CD8 (**a**) and CD4 (Foxp3^-ve^) (**b**) T cells in control and IT-treated mice. **c**-**d** Pie charts depicting central memory (white) vs effector/effector memory (black) frequency in the CD44^high^ sub-population in CD8 (**c**) T cells and CD4 (**d**) T cells; frequencies of CD44^high^ depicted within pie slices for given population. (**e**-**f**) Frequency of effector/effector memory (**e**) and central memory (**f**) CD8 (left panels) and CD4 (Foxp3^-ve^) (right panels) T cells in various organs from control or anti-CD40/IL2-treated mice. These data are representative of 4-5 independent experiments with 3 mice per group. Data are presented as mean ± SEM

Previous studies have shown that memory phenotype cells (CD44^high^) are the main cell type expanding following stimulatory immunotherapies [6]. To better understand the composition of CD4 and CD8 T cells across various organs, we evaluated their memory phenotype status in each organ following IT. At rest, the CD44^high^ population of CD8 T cells in the lymphoid organs was predominantly T_CM_ (>90%) whereas in the peripheral organs, it was a combination with ~60% T_CM_ (Fig. 3a, c, e,-f). In general, IT results in an overall expansion in the CD44^high^ frequency across all organs. The T_CM_ frequencies were either unchanged or slightly increased, while the T_E/EM_ populations significantly expanded (Fig. 3e-f) from ~10% to 30% in the lymphoid organs and, impressively, from ~30-85% in the peripheral organs.

Within the CD44^high^ population of the CD4 T cells, resting mice were more heavily skewed towards the T_E/EM_ phenotype with approximately 60-70% in the lymphoid and 75-95% in the peripheral tissues (Fig. 3b, d). As occurred in the CD8 T cells, following IT the CD44^high^ proportion expanded but due to the fact that it was so heavily skewed to the T_E/EM_ phenotype across all organs in resting mice, the frequencies of CD4 T_E/EM_ were largely consistent across all organs in IT-treated mice (Fig. 3e). The T_CM_ CD4 frequencies remained relatively low and consistent across all organs, both pre- and post-IT (Fig. 3f).

### Expression of activation markers in CD4 and CD8 T cells is dependent upon location and memory phenotype

In addition to differences in proliferation and apoptosis, we have also routinely noticed that CD4 and CD8 T cells differentially upregulate activation and inhibitory molecules following IT. The most notable example of this being PD-1 which, based on studies focusing on secondary lymphoid organs (spleen and LN), was preferentially upregulated on CD4 and not CD8 T cells and thought to likely be involved in the preferential AICD process that occurred in CD4 but not CD8 T cells following IT [7]. Another example would be the preferential upregulation of NKG2D on CD8 T cells but not CD4 conferring bystander-induced lytic capability following strong cytokine exposure to the memory CD8 subset. Previous studies by our lab as well as data presented in Fig. 3 have shown that among both CD4 and CD8 T cells, the primary cells that actively proliferate and respond to IT are the CD44^high^ memory phenotype cells [6]. Therefore, we next focused on this population.

**Fig. 4:**
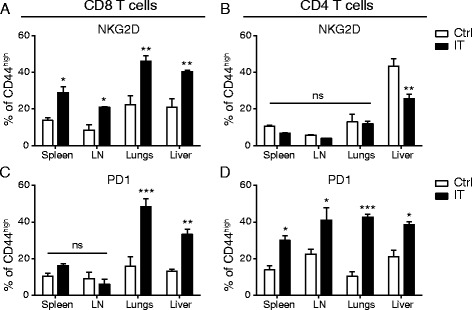
Differential expression of activation and inhibitory markers in CD8 T cells dependent upon location. Mice were treated with anti-CD40/IL-2 immunotherapy and assessed for various immune parameters on day 12 of treatment in lymphoid (spleen or LN) or peripheral (lungs or liver) organs. Percentage NKG2D^+^ (**a**-**b**) and PD-1^+^ (**c**-**d**) of CD8 (**a**, **c**) and CD4 (Foxp3^-ve^) (**b**, **d**) T cells across various organs. Pie graphs depicting CD8 EM/CM of each organ under given treatment conditions/organ. These data are representative of 2-4 independent experiments with 3 mice per group. Data are presented as mean ± SEM. Statistics were derived using ANOVA with Bonferroni’s post-test, *P < 0.05, **P < 0.01, ***P < 0.001

In the CD44^high^ population, it has been shown that the proliferating CD8 T cells fail to upregulate markers consistent with activation by an antigen specific stimulus such as CD25 and PD-1, yet upregulate markers that allow them to acquire a bystander phenotype, namely NKG2D, conferring the ability to act more in an NK-like, antigen unrestricted manner. Conversely, CD44^high^, proliferating (Foxp3^neg^) CD4 T cells disproportionately upregulate PD-1 (in contrast to CD8 T cells and Foxp3+, regulatory CD4 T cells) which we have suggested allows them to be preferentially targeted for induction of apoptosis [8]. Consistent with these previous reports, we observed similar phenotypes among splenic and lymph node resident, IT-treated CD44^high^CD8+ T cells, which significantly upregulated NKG2D but not PD-1 (Fig. 4a, c), and CD44^high^CD4+ T cells, which robustly upregulated PD-1, yet not NKG2D (Fig. 4b, d). When we assessed the same phenotypic markers in the T cell populations resident to peripheral, non-lymphoid organs, the CD44^high^CD8+ T cell phenotype was considerably different from that of those resident to the secondary lymphoid organs. While CD44^high^CD8+ T cells resident to the lungs and liver were still NKG2D^+^CD25^neg^ (Fig. 4a, c), the frequency of NKG2D+ cells in this population appeared to increase from 20-30% in the lymphoid organs to 40–50% in the peripheral organs (Fig. 4a). Furthermore, in contrast to lymphoid organs where PD-1 expression was unchanged, PD-1 expression was increased significantly in both the lungs and liver following IT in the CD44^high^CD8+ population (Fig. 4c). Conversely, CD44^high^CD4 T cell phenotype was remarkably similar to spleen and lymph node CD4 T cells across all organs (Fig. 4b, d) with comparable expression of PD-1, and minimal upregulation of NKG2D. CD25 was not upregulated in CD4 or CD8 T cells at any site (data not shown). This was unexpected because we have previously suggested that the differential expression of PD-1 was likely the underlying mechanism of the differential induction of apoptosis between CD4 and CD8 T cells following strong, immunostimulatory IT regimens. Yet, in the peripheral organs, CD4 T cells continue to be disproportionately affected by apoptosis despite the fact that PD-1 expression is comparable between CD4 and CD8 T cells. This pattern that emerged was also interesting because the increased activation marker expression in the periphery appeared to directly correlate with T_E/EM_ predominance, particularly in the case of PD-1.

**Fig. 5:**
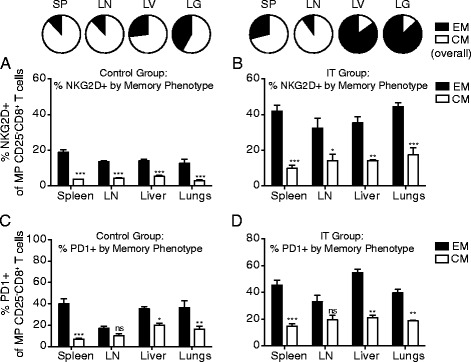
Differential phenotypes of CD8 T cells by location correlates with enhanced expansion and activation marker upregulation on the effector/effector memory T cell phenotype. Mice were treated with anti-CD40/IL-2 immunotherapy and assessed for various immune parameters on day 12 of treatment in lymphoid (spleen or LN) or peripheral (lungs or liver) organs. Frequencies of NKG2D+ (**a**-**b**) and PD-1+ (**c**-**d**) in CD25^neg^CD44^high^CD8^+^ T cells in control (**a**, **c**) and anti-CD40/IL-2 (**b**, **d**) treated mice as stratified by T_CM_ (CD62L+, white) and T_E/EM_ (CD62L-, black). These data are representative of 2-3 independent experiments with 3 mice per group. Data are presented as mean ± SEM. Statistics were derived using ANOVA with Bonferroni’s post-test, *P < 0.05, **P < 0.01, ***P < 0.001

It has recently been shown that circulating T_E/EM_ cells express elevated levels of PD-1 in resting humans [15]. Therefore, we hypothesized that CD8+ T_E/EM_ cells may be preferentially expressing these activation markers over CD8+ T_CM_ resulting in differential frequencies of CD44^high^CD8+ T cells expressing activation markers in secondary lymphoid and peripheral organs following IT. Therefore, we evaluated NKG2D and PD-1 expression on CD8 + CD44^high^CD25^neg^ T_E/EM_ and T_CM_ cells across all organs in resting and IT-treated mice. In control mice, both NKG2D (Fig. 5a) and PD-1 (Fig. 5c) were expressed at a higher frequency on the T_E/EM_ subset of the CD8^+^CD44^high^CD25^-^ population. However, the overall frequency of the T_E/EM_ population among CD8^+^ T cells in resting mice is relatively low compared to T_CM_ (pie charts Fig. 5a), therefore, overall the expression of both PD-1 and NKG2D is predominantly low (Fig. 4) as T_CM_ makes up the majority of CD8+ T cells at rest. In the immunotherapy treated mice, both the NKG2D and PD-1 expression were increased across all organs (Fig. 4). Once again, both NKG2D (Fig. 5b) and PD-1 (Fig. 5d) were expressed more highly on the T_E/EM_ CD8+ T cells than the T_CM_ CD8+ T cells. In the lymphoid organs, where the T_E/EM_ population expanded compared to control, it was still significantly less than CD8+ T_CM_ cells (pie charts, Fig. 5b) resulting in less significant expansions at these sites. Contrary to lymphoid organs, CD8+ T_E/EM_ cells made up the majority of the peripheral organs assayed (pie charts, Fig. 5b) thereby making the overall expression of NKG2D and PD-1 significantly higher at these sites. Again it is important to note that in the lymphoid organs of immunotherapy-treated mice, the overall expression of both activation markers was significantly lower than in the peripheral organs due to the T_CM_ skewing in the lymphatics over the periphery in the CD8 population. The expression levels didn't vary greatly among T_E/EM_ from different organs (there were no significant differences between lymphoid and peripheral organs) within the same treatment groups but did generally increase in the IT-treated compared to control, a trend that was more significantly pronounced with NKG2D than PD-1 (Fig. 5). In contrast, T_CM_ activation marker expression remained relatively constant not only among organs from mice within a treatment group, but between control and IT-treated groups as well (Fig. 5a-b). Taken together, these data suggest that the constitution of the memory/activated pool (T_CM_ vs T_E/EM_) weighs heavily upon the phenotype of the activated T cell population, particularly with CD8 T cells as their constitution varies greatly between lymphoid and non-lymphoid organs.

### Assessment of T cells from patients receiving high dose systemic immunostimulatory therapy

**Fig. 6:**
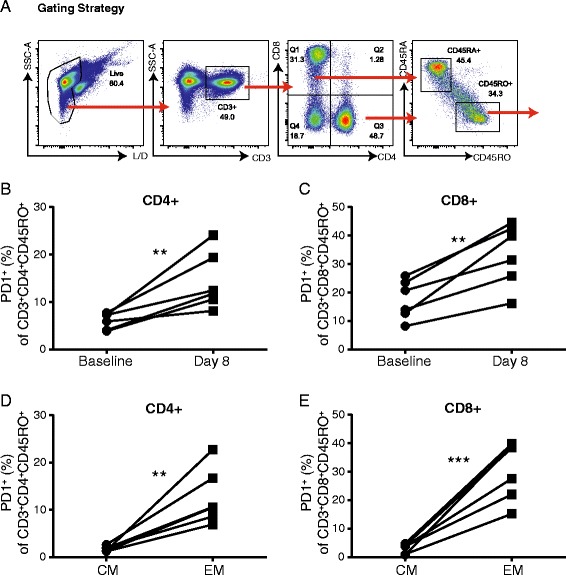
Effector/effector memory T cells from human T cells undergoing IL-2 therapy express upregulated PD-1. PBMCs were isolated prior to therapy and on day 8 of treatment from patients undergoing high dose systemic IL-2 therapy for melanoma. PBMCs were assessed for T cell subset expression of PD-1 by flow cytometry. **a** Representative gating strategy for staining of human PBMCs. **b**-**c** Frequency of PD-1 expression on memory CD4 (**b**) and CD8 (**c**) T cells. (**d**-**e**) Frequency of PD-1 expression on central (CD45RO + CD62L+) and effector memory (CD45RO + CD62L-) subsets in CD4 (**d**) and CD8 (**e**) T cells. Six patient samples were included in this data set. Data are presented as mean ± SEM. Statistics were derived using Student’s T test, *P < 0.05, **P < 0.01, ***P < 0.001

Next we wanted to assess whether these results translated into human patients receiving immunostimulatory therapies for cancer. There are currently no trials assessing combination agonistic anti-CD40 with recombinant human IL-2 however we have routinely compared our combination therapy to other systemic immunostimulatory treatments including high dose TLR agonists and high dose systemic cytokine therapies and shown similar phenotypic and functional changes to T cells as are observed in our preclinical model [5, 16]. To assess whether patients in the clinic displayed similar changes in surface marker expression, we collected peripheral blood mononuclear cells (PBMCs) from metastatic melanoma patients undergoing systemic high dose IL-2 therapy. Patients received 6x10^5 IU/Kg every 8 h for a planned total of 14 doses. PBMC samples were collected one day prior to the start of therapy (baseline) or on day 8 of the first cycle of therapy (day 8) to assess T cell phenotype. Comparing baseline and day 8 samples, there was a significant increase in PD-1+ memory phenotype (CD45RO+) cells in both the CD4 and CD8 T cell subsets following high dose IL-2 therapy (Fig. 6a-c). When this population was further broken down into central memory (CD62L+) and effector/effector memory (CD62L-) at the day 8 time point, the effector/effector memory subset expressed significantly higher PD-1 expression than the central memory subset (Fig. 6d-e). Together, these data correlate with what was observed in murine studies suggesting that these data are applicable to human studies and may be an indicator of what is occurring locally.

## Discussion

Our previous studies have shown that treatment with systemic immunostimulatory therapies for cancer results in preferential induction of apoptosis in the CD4 T cell population in an IFNg-dependent mechanism [7]. We further built upon that study suggesting that it was the preferential induction of PD-1 on the CD4 T cell effector population (as opposed to regulatory CD4 or CD8 T cells) that was responsible for their bias to activation induced cell death [8]. This study suggests that this may not actually be the case in that PD-1 expression in CD8 T cells in peripheral organs is actually significantly elevated, much more so than CD4 T cells in the lymphoid organs following IT, yet they continue to expand without inducing elevated levels of apoptosis. This would suggest a role for PD-1 that is more in line with its function as an acute activation marker in activated cells [17, 18] rather than its function in inducing apoptosis as occurs during exhaustion [19–21]. This study again underscores the necessity for evaluating multiple organ sites when drawing conclusions, particularly during murine studies where this can be easily done.

Given that the majority of studies evaluating immune parameters do not directly examine target tissues but examine peripheral blood or take biopsies from lymphoid organs (for example, whole spleens in mice or LN biopsies in humans), this study suggests that we are not getting accurate measures of what is truly occurring at important effector sites when looking at frequencies and counts of overall populations. For example, PD-1 expression was not significantly upregulated (less than ~20%) in the spleen and lymph nodes of immunotherapy treated mice but was greater than ~40% in the lungs and liver (Fig. 4). These data underscore the need to consistently evaluate immune parameters across as many organ sites as possible in order to get an accurate picture of what is occurring in the organism as a whole. Indeed, in many clinical trials involving immunotherapies for cancer, it has proven difficult to pin point correlates of objective responses in peripheral blood. In this study, correlations were drawn with the composition of the memory phenotypes of different T cell populations with the actual activation marker phenotypes observed in the population as a whole (Fig. 5). In general, we saw that the T_E/EM_ population, while smaller overall in the lymphoid organs, was relatively accurate as to the overall phenotype of the activated T cells at effector sites (peripheral organs). While many human clinical trials aren't able to fully evaluate effector sites, this may become a powerful tool moving forward as it may prove useful to look for correlates of objective responses focusing on the T_E/EM_ phenotype which appears to mimic the effector site more closely (assuming that the effector site is a not a lymphoid organ). Indeed, a recent study by Gros et al. showed that *only* the T cells expressing PD-1 (and by extrapolation of work presented within this paper likely of the T_E/EM_ phenotype) in the peripheral blood shared TCR specificity with tumor infiltrating lymphocytes found in the tumor [22]. Additionally, while the data is not shown in the current study, the phenotype of T cells within tumors following systemic immunostimulatory therapies such as anti-CD40/IL-2 or systemic high dose IL-2 have previously been extensively characterized [7, 10]. In the case of these systemic immunostimulatory regimens, it is important to consider T cell phenotypes without tumor burden as the overwhelming majority of the T cells activated in tumor bearing studies are antigen non-specific bystander memory T cells. These non-specific bystander CD8 T cells have a prominent role in tumor clearance as has been previously shown [5, 6]. In order to reconcile this, however, we show that the phenotype of T cells in the tumor is comparable to that in the tissues thus highlighting the relevance of using tissues that are often targets of metastatic sites (i.e. liver and lungs) by demonstrating T cells phenotypes from the MINO tumor model as well as lungs and spleen (Additional file 1: Figure S1).

Altogether, we show that following cancer immunotherapy we can observe a similar population of bystander activated CD8 T cells whose expression of different key activation markers varies greatly depending upon their location within the body and the composition of the memory T cell pool at that location. Following activation, bystander memory T cells are generated from 1) central memory T cells and/or 2) effector memory T cells, with the effector memory T cells being phenotypically similar to effector T cells. The locations of these cells vary with subset (4 vs 8) and memory phenotype (naïve vs central memory vs effector memory). In general, the memory proportion of the CD4 subset is more heavily T_E/EM_ skewed within the lymphoid organs at rest comprising a CD4 population made up predominantly of naïve and E/EM cells [12, 13]. In contrast, the memory proportion of the CD8 subset is more heavily T_CM_ skewed within the lymphoid compartment at rest comprising a CD8 population made up predominantly of naïve and central memory cells [12, 13]. Contrary to differential distribution within the lymphoid compartment, the memory populations of both the CD4 and CD8 subsets in the peripheral, tissue resident populations is largely effector/effector memory skewed. [14] Our studies revealed that CD8 T_CM_ had relatively lessened expression of key activation markers such as NKG2D and PD-1 whereas CD8 T_E/EM_ had relatively heightened expression of the same markers (Fig. 5). Therefore, the composition of the memory pool at different sites weighed heavily on the overall expression of those markers in the memory pool. This made it appear as if the expression of these key markers may be changing at different sites when in fact it was the composition of the bystander activated population (T_CM_ vs T_E/EM_) that was actually altered.

Finally, expression of activation markers and T cell memory phenotype distribution changes over the course of a lifespan with variables such as age, body fat content, and pathogen status (such as SPF vs non-SPF), among other things. Now that we are beginning to appreciate the impact of each of these conditions on responses to infectious disease, responses to immunomodulatory treatments, and even the maintenance of homeostasis (as compared to young, non-obese, SPF counterparts), it is important to understand and assess how differences at baselines can affect outcomes across all organs. For example, obese and aged mice generally express elevated PD-1 [23] (and manuscript in progress) on T cells and have been shown to have a skewed memory phenotype [23–25]. What has not been thoroughly assessed in these mice is whether the PD-1 is predominantly on the T_E/EM_ populations that the T cells are skewed into which this study would suggest may be the underlying root of the distorted PD-1 expression. In conclusion, we have presented data herein illustrating the differences in activation marker expression based on memory phenotype which varies between lymphoid and non-lymphoid organs both at rest and during an active immune response. These data underscore the necessity to thoroughly investigate both lymphoid and peripheral sites before drawing conclusions based on cell phenotype and function.

## Conclusions

In summary, we show that there can be significant differences in T cell phenotype based on location of the cells within lymphoid organs or at peripheral sites. In particular, the T_E/EM_ subset of T cells in lymphoid organs more accurately reflect the phenotype of T cells at the peripheral sites. These data highlight PD-1 expressing and/or T_E/EM_ subsets of T cells in circulation as more representative of cells at immune sites and underscore the importance of valuation both in lymphoid as well as target organs when making determinations about immune status.

## Additional file

Additional file 1: Figure S1. Differential T cell phenotypes in lymphoid vs primary tumor and metastatic sites following anti-CD40/IL-2 immunotherapy. MIN-O mice were developed as previously described. (9). Once tumors were palpable within MIN lesions, mice were treated with anti-CD40/IL-2 immunotherapy. On day 9 of therapy, mice were taken down to assess T cell phenotypes in the spleen, lungs, and tumor. (A) Survival of control and anti-CD40/IL-2 (40/2) treated MIN-O mice. (B-C) Representative dot plots of CD8+ T cell phenotypic analysis in control and immunotherapy treated MIN-O mice. Data are representative of 1-2 independent experiments. (PDF 882 kb)

## Abbreviations

BrdU: Bromodeoxyuridine
Foxp3: Forkhead box p3
IFNg: Interferon gamma
IL-2: Interleukin-2
IT: Immunotherapy
IU: International units
Kg: Kilogram
KLRG1: Killer cell lectin like receptor subfamily G member 1
LN: Lymph node
MHC: Major histocompatibility complex
MIN-O: Mammary intraepithelial neoplasia outgrowth
NKG2D: Natural killer group 2D
PBMCs: Peripheral blood mononuclear cells
PD-1: Programmed death 1
SPF: Specific pathogen free
T_CM_: central memory T cell
TCR: T cell receptor
T_E/EM_: effector/effector memory T cell
TLR: Toll like receptor
