# Supplementary figures and images for: Differential phenotypes of memory CD4 and CD8 T cells in the spleen and peripheral tissues following immunostimulatory therapy
Source: J Immunother Cancer. 2017 Apr 18;5:33. doi: 10.1186/s40425-017-0235-4 (PMC5394626; doi:10.1186/s40425-017-0235-4)

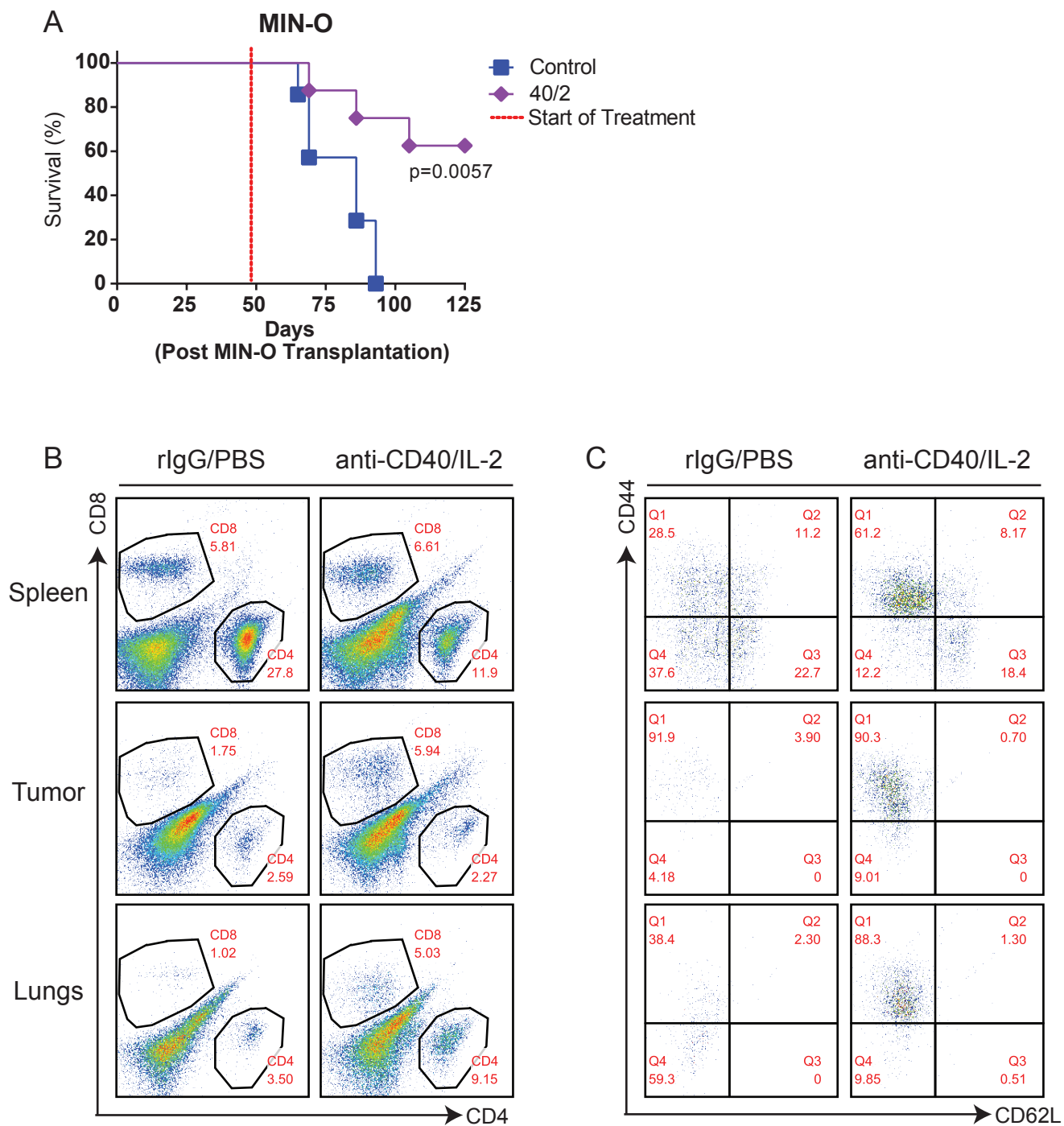

**Supplemental Figure 1**

Supplement: Additional file 1: Figure S1. — Differential T cell phenotypes in lymphoid vs primary tumor and metastatic sites following anti-CD40/IL-2 immunotherapy. MIN-O mice were developed as previously described. (9). Once tumors were palpable within MIN lesions, mice were treated with anti-CD40/IL-2 immunotherapy. On day 9 of therapy, mice were taken down to assess T cell phenotypes in the spleen, lungs, and tumor. (A) Survival of control and anti-CD40/IL-2 (40/2) treated MIN-O mice. (B-C) Representative dot plots of CD8+ T cell phenotypic analysis in control and immunotherapy treated MIN-O mice. Data are representative of 1-2 independent experiments. (PDF 882 kb) [file 40425_2017_235_MOESM1_ESM.pdf]
